# Developing a scale to explore self-regulatory approaches to assessment and feedback with academics in higher education

**DOI:** 10.3389/fpsyg.2024.1357939

**Published:** 2024-03-25

**Authors:** Carol Evans, William Kay, Sheila Amici-Dargan, Rafael De Miguel González, Karl Donert, Stephen Rutherford

**Affiliations:** ^1^Cardiff University, Cardiff, United Kingdom; ^2^School of Biosciences, Cardiff University, Cardiff, United Kingdom; ^3^School of Biological Sciences, Faculty of Health and Life Sciences, University of Bristol, Bristol, United Kingdom; ^4^Faculty of Education, University of Zaragoza, Zaragoza, Aragon, Spain; ^5^European Association of Geographers (EUROGEO), Brussels, Belgium

**Keywords:** self-regulatory assessment and feedback practices, higher education, academics, professional development, scale reliability

## Abstract

**Introduction:**

Students need to acquire high level self-regulatory skills if they are to be successful within higher education, and academics need support in facilitating this. In this article we explore how the current research gap between knowledge of self-regulatory assessment and feedback (SRAF) practices, and academics’ professional training in it can be bridged.

**Methods:**

SRAF tools were used with academics to explore their understandings of and training needs in SRAF; central to this work was the development of a SRAF scale. We consider the value of such tools in supporting academics’ professional development needs in SRAF. The reliability and validity of the SRAF scale was tested using exploratory factor analyses (EFA).

**Results:**

Iterative EFA resulted in a 17 item support required SRAF scale (SR). Two underpinning factors: *Creating the Conditions for SRAF*, and *Supporting Students’ SRAF Skills Development* were identified. The reliability of the instrument supported its primary use as a tool to facilitate academics’ professional development in fostering students’ self-regulatory skills.

**Discussion:**

Our findings highlight the importance of supporting academics in developing strategies to maximize students’ metacognitive skills and motivation in assessment and feedback, contingent on effective assessment design. Such professional development needs to be mindful of individual and contextual factors impacting academics’ access to, and confidence and competence in, using SRAF in practice. This research is important in highlighting potential disconnects between where academics’ focus their attention in assessment, and what is known to have most impact on student learning success. The SRAF tools have considerable potential in supporting translation of theory into practice as part of sustained professional development for academics in higher education.

## 1 Introduction

The importance of supporting students’ self-regulatory learning (SRL) skills development in impacting their achievements in higher education is well known (Hayat et al., 2020; Büchele, 2023; Hattie, 2023; Evans and Waring, 2023a). Supporting academics in providing such skills support to students is challenging given that training in self-regulatory practices is significantly underrepresented in professional development provision for academics in higher education (Ruiz and Panadero, 2023). Translation of knowledge on effective self-regulatory assessment and feedback (SRAF) into practice is limited by the lack of guidelines available to academics on how to do this well (Honig and Coburn, 2007; Jansen et al., 2019). Academics need to know how to support students’ SRL skills development, and as part of this, they need better understanding of the relationships between learner characteristics and personal goals, and cognitive, metacognitive and emotional regulatory processes, and how these impact learning.

As identified above, supporting students’ SRL skills development is essential given the relationship between self-regulatory capacity and student achievement (Schneider and Preckel, 2017; Lin et al., 2022). Students enter higher education with varying levels of self-regulation. Of significant importance is that students’ abilities to self-regulate can be developed (Vosniadou, 2020), while accepting that some students are more capable of self-regulatory flexibility than others (Kozhevnikov et al., 2014). To support students’ academic development, and to utilize resources most effectively it makes sense for academics to focus on those SRL skillsets that are most implicated in student success (Dinsmore, 2017). As noted by Russell et al. (2022), academics’ self-regulation plays an important part in how academics’ support student SRL skills development. For academics to be able to do this effectively, they need to be aware of their own SRL skillsets, identify and focus on those high level SRL skills students most need within a specific context, and model these skillsets confidently with their students.

However, the complexity of the self-regulation construct makes translation of it into assessment and feedback practice in higher education difficult given that is an umbrella concept (Panadero, 2017) comprising many different variables and approaches with different theoretical underpinnings. Evans et al. (2021, p. 10) in exploring the multi-faceted nature of self-regulated learning (SRL) define it as:

a learner’s ability to regulate his/her learning in different contexts… SRL can be viewed as a construct, a process and an ability that can be developed… SRL may comprise state (approaches developed in response to a specific context) and trait elements (established patterns of working that are consistent across contexts).

Zimmerman (1998, p. 329) argued that “students can be described as self-regulated to the degree that they are metacognitively, motivationally, and behaviorally active participants in their own learning process”, and that “students’ learning must involve the use of specified strategies to achieve academic goals on the basis of self-efficacy perceptions”. Self-efficacy in this context refers to students’ perception of their own abilities to manage the learning process effectively, and achieve their desired goals. In exploring the structure of self-regulation, Zimmerman and his contemporaries discuss the recursive stages involved in managing a task such as *forethought* (planning and goal-setting), th*e performance phase* (selection of appropriate strategies to complete a task and ongoing monitoring and review to maintain motivation and adjust strategies as necessary), and a *self-reflection phase* (involving self-evaluation of effectiveness and reframing as necessary in pursuit of goals). In all these phases metacognitive (understanding of which strategies to use), cognitive (how individuals make sense of and process information) and affective strategies (management of emotional aspects of learning) are required (Evans et al., 2021).

In this work, we were particularly interested in the metacognitive skills students deploy in assessment and feedback while acknowledging the interdependence of these with cognitive and affective strategies. The mediating nature of task requirements (e.g., nature of assessment), the context (e.g., extent to which the design of assessment requires and values students’ acquisition of high level self-regulation skills), and individual characteristics (e.g., self-efficacy and motivation) make it difficult to ascertain how best to support students in choosing the most appropriate SRL strategies and using them well (Dresel et al., 2015). Jansen et al. (2019) concluded from a review of 142 studies that academics need to support students’ engagement in SRL activities as well as their achievement; they argue that the lack of significant moderators of the effects of SRL interventions makes it difficult to provide concrete design guidelines for such SRL interventions. In providing more specific guidance, Hattie et al. (1996) recommended that training should be in context, and use tasks within the same domain as the target content, and promote a high degree of learner activity and metacognitive awareness. Evans and Waring (2023a) have gone further in articulating the key elements of a SRAF approach to support translation of research into practice through encouraging academics to articulate what those high level self-regulatory skills are that they want students to develop, and by providing a route map of how to build participatory assessment designs that provide the conditions in which development of these skills can flourish.

In this article we describe a pilot exploratory project developed to support better understanding of SRAF in practice, conscious of the relative lack of research on supporting academics’ professional development in SRAF in higher education research. This research is important given that academics’ knowledge of self-regulatory approaches impacts the quality of assessment design (Dörrenbächer and Perels, 2016; Peeters et al., 2016), and the fact that professional development for academics in this area is in its infancy (Evans and Waring, 2023a; Dinsmore et al., 2024).

This research is situated within the context of developing tools to support academics’ translation of SRAF into practice as part of an international Erasmus+ research funded project. This project used an established, research-informed assessment framework (EAT). The Equity, Agency and Transparency (EAT) framework was chosen to explore how best to support academics’ access to, and effective use of relevant SRAF approaches given its underpinning theoretical framing around agentic, inclusive, and self-regulatory approaches to assessment and feedback. This theoretical and conceptual framework (Evans, 2016, 2022) synthesizes what is known about effective assessment and feedback (Evans, 2013) and integrates this with an understanding of self-regulatory learning approaches and individual differences in learning. EAT was developed from extensive systematic review of the literature and evolution of the framework with staff and students across disciplines and higher education institutions. Its visual form is that of a wheel which guides academics to consider 12 core entry level questions about how they support students’ SRAF, and asks students how they engage with SRAF. There is a vast body of resources to support learners (academics and students) to consider how best to develop and evaluate the effectiveness of their approaches to SRAF using a research-informed approach. A key strength of the approach is it can be adapted to any context and any level of analysis (individual, course, discipline, faculty, institution) (Evans et al., 2022a).

Conceptually, EAT (Evans, 2022) highlights the central role of academics in designing assessment environments that support students’ SRL skills development in partnership with them. Partnership involves active engagement with students in decision-making processes about assessment and feedback to support co-ownership of assessment, dependent on supporting students’ skills development and confidence in being able to step up to take a more central role in the assessment and feedback process, and includes defining the limits of their engagement. Through partnership in assessment, it is argued that student agency is increased, creating opportunities for students to impact the quality of assessment, which in turn enhances the conditions to support SRL skills development (Evans and Waring, 2021).

Emphasis on how conditions are created to promote student ownership and agency in assessment is a central element of our SRAF pedagogical framing and aligns with Bandura’s (1986, 2001) idea of agency in how individuals deliberately guide their behavior (the actions they choose and how they execute them in pursuit of goals), and Reeve’s (2013) notion of agentic engagement in how individuals are empowered by their environment so they are able to leverage change within it. It also requires academics to be discerning in selecting what high level self-regulatory skills they wish to focus on related to their specific context.

In this article we: (i) consider what a SRAF approach is, and the key dimensions of it implicated in student learning in higher education, (ii) explore what SRAF support academics want to enhance their assessment practice, and in relation to their perceived use of SRAF, and (iii) consider the implications for the development of SRAF professional development in higher education from using SRAF tools with colleagues. To address these questions, we firstly, explore the context of SRAF in higher education. Secondly, we describe pedagogical tools developed to support understanding of SRAF including the development of a scale to explore academics’ perceived frequency of use of SRAF practices, and associated professional development needs, drawing on the model developed by Dinsmore et al. (2024). Thirdly, we model the outcomes from our work with academics on using SRAF tools, and explore the implications of these findings for enhancing SRAF professional development in higher education.

## 2 Developing a SRAF pedagogy

Evans and Waring (2023a,b) coined the term SRAF pedagogies to refer to assessment and feedback practices that focused on the systematic development of students’ SRL skillsets, and critical evaluation of them in practice. In their approach SRL is embedded within all aspects of assessment design and emphasis is placed on supporting students’ knowledge, skills and confidence in their ability to choose the most appropriate learning strategies and to use them effectively within a specific context (i.e., attuned to disciplinary and professional needs). According to Evans and Waring (2023a, pp. 11–12).

SRAF considers learner characteristics and personal goals, and how cognitive, metacognitive, and emotional regulatory processes come together to support learning. Of critical importance is the degree of alignment between academics’ and students’ perceptions of quality in impacting improvements in learning … A key emphasis in the design of self-regulatory assessment has to be on how we maximize the opportunities for students to gain an understanding of quality for themselves.

There are many potential permutations of SRAF pedagogies which may have different emphases depending on different theoretical perspectives on SRL, and in relation to how academics perceive the role of students in the process. Evans and Waring (2023a) highlight the importance of effective assessment design in creating the necessary conditions for SRAF to support students’ agentic engagement with assessment and feedback. Agentic engagement involves students’ abilities to evolve their learning context to address their assessment needs. In providing explicit guidelines on SRAF, they argue that academics need to start by articulating what the core SRL skills they want students to acquire within their discipline are.

In drawing together research on SRAF, Evans and Waring (2023a) argue that emphasis should be on ensuring the assessment context supports SRL development, and provides focused skills training by attending to the following:

- Embedding SRL skills development within discipline-specific contexts (Hattie et al., 1996).
- Ensuring SRL skills development is integrated into all aspects of assessment and feedback design (Evans, 2016, 2022).
- Addressing academics’ and students’ conceptions of their roles in assessment and feedback to support student agency and autonomy (Vermunt and Donche, 2017).
- Focusing activities to support alignment of academics’ and students’ conceptions of quality.
- Working with students to support their engagement as co-constructors of assessment and feedback practices to support internalization of standards (Hattie et al., 1996; Simper, 2020; Nicol, 2022).
- Making explicit what the core high level SRL skills are that students need to be successful within a course (Evans, 2016, 2022; van Merrienboer and de Bruin, 2019).
- Focusing on the development of high-level SRL skills that have the most impact on learning outcomes (e.g., motivational and metacognitive) (Dinsmore, 2017; Panadero, 2017; van Merrienboer and de Bruin, 2019; Dekker et al., 2023).
- Providing repeated opportunities for students to observe, emulate, apply and evolve self-regulation strategies that are most relevant to the contexts they are working in (Zimmerman and Kitsantas, 2005; Dunlosky et al., 2013).
- Using data and technologies with academics and students to support their understanding of their learning, and the implications of different teaching and learning approaches on outcomes (Tempelaar et al., 2021; Hattie, 2023).
- Acknowledging and addressing the increasing role of digital including artificial intelligence (AI) literacy in self-regulatory skills development (Istifci and Goksel, 2022; Krempkow and Petri, 2022).
- Placing emphasis on high quality professional development in SRAF supported by high quality research design including evaluation processes (Panadero, 2023).

## 3 Theoretical framing

### 3.1 SRAF skills development

In developing SRAF professional development frameworks and tools, as previously identified, we drew on the EAT assessment and feedback framework (Evans, 2016, 2022) given its strong integrated theoretical frame. EAT brings together constructivist, socio-cultural and socio-critical theories in supporting effective self-regulatory assessment and feedback (Evans, 2013) with understanding of student approaches to learning (SAL) (Vermunt and Verloop, 1999), and agentic engagement (Reeve, 2013).

EAT aligns with socio-cognitive (Bandura, 1986, 1991, 2001; Pintrich, 1989, 2004; Zimmerman, 2001; Zimmerman and Campillo, 2003) and information processing self-regulation models (Winne and Hadwin, 1998; Winne, 2001). Socio-cognitive models emphasize the role of interaction with others in impacting learning behaviors, and information processing models focus on how individuals make sense of information, and the cognitive, metacognitive, and affective processes inherent in this.

**Figure 1:**
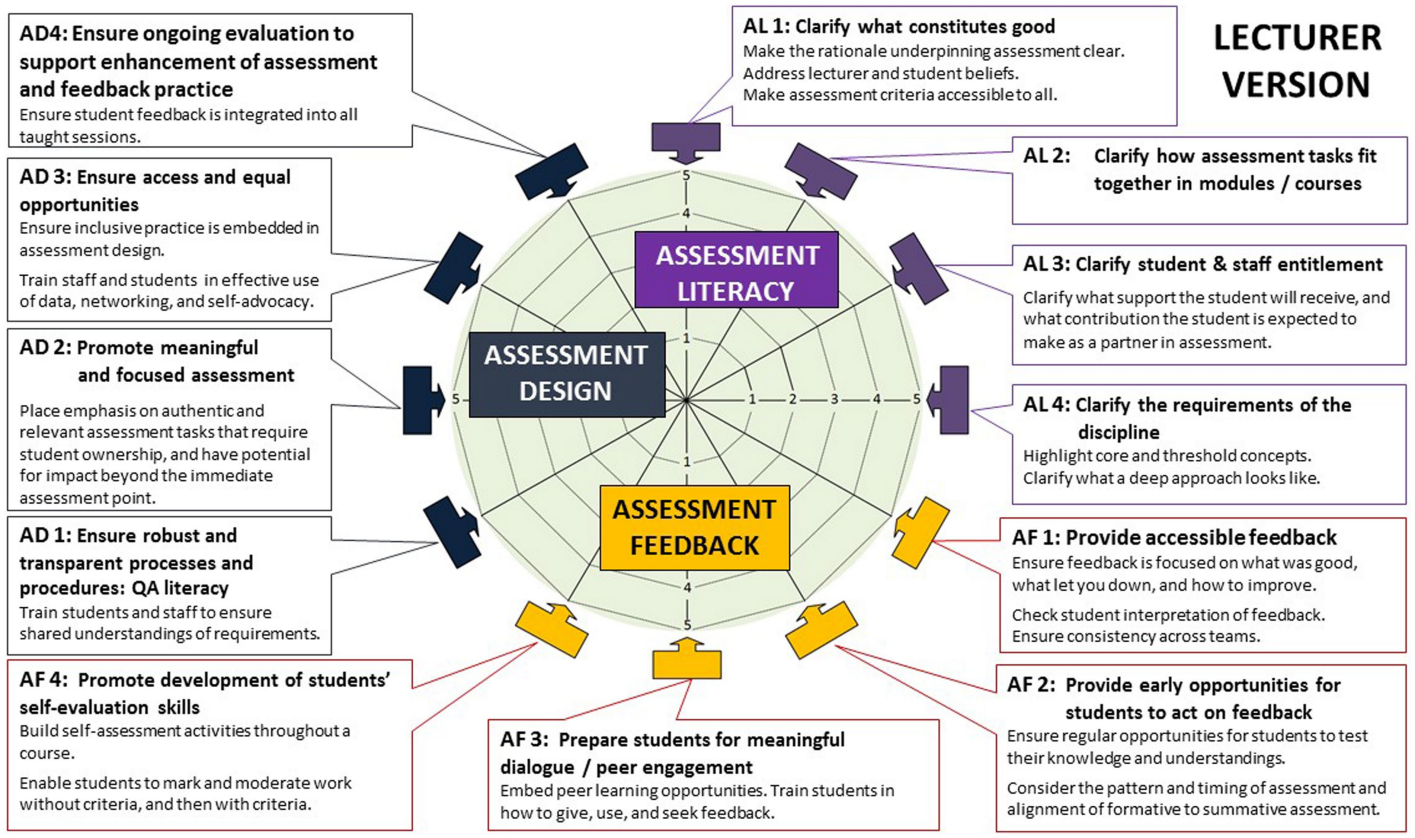
The dimensions of the EAT Framework (Evans, 2022).

In Figure 1, EAT portrays effective assessment and feedback practices (Evans, 2013) as 12 interconnected sub-dimensions of assessment literacy (AL), assessment feedback (AF), and assessment design (AD). The EAT sub-dimensions are all highly integrated, in that actions taken in one aspect of assessment and feedback practice have an impact on others. Academics are asked to consider how they engage students in supporting their self-regulatory development in each of these sub-dimensions of practice as integral to the focus of the model on ensuring student access to assessment and feedback and their agentic engagement with it. The quality of assessment design and a supportive institutional context are important in providing the conditions to support SRAF development for students and academics, respectively. The relational dimension of SRL involves being able to utilize one’s own skills effectively, and gain support from others in the realization of one’s learning goals. Agency and engagement are identified as essential in supporting SRL skill development and achievement (Boud and Molloy, 2013; Evans, 2013). Our approach recognizes the combined influence of individual dispositions, metacognitive, cognitive, and affective strategies, and contextual affordances and barriers in impacting learners’ management of assessment and feedback (Vermunt and Verloop, 1999).

### 3.2 SRAF skills development

Our SRAF approach considers how learners acquire competencies, the importance of individuals’ and teams’ conceptions and beliefs on this process (Bembenutty et al., 2015), and awareness of the different ways in which learners process information (Waring and Evans, 2015). The importance of explicit teaching of SRL skills is intrinsic to this approach, while also acknowledging that some individuals are capable of higher level SRL skills development than others, especially in relation to metacognitive flexibility (Kozhevnikov et al., 2014). Emphasis is also on supporting quality and conditional use of strategies; using strategies effectively and selecting the most appropriate ones for a given task (Dinsmore, 2017).

From a self-regulatory process perspective, key metacognitive skillsets required in managing assessment tasks include *accuracy in interpreting the requirements of a task* and *meta-memory in ascertaining what you know*, and how you can use this knowledge to support task completion. In planning an appropriate approach to manage assessment *the quality and nature of goals* (Dent and Koenka, 2016) and *contextual regulation* (being able to read the context well in knowing where and who to get support from and how to use such support well) are important. *Monitoring accuracy* is dependent on *effective use of cues* coming from the task itself, the task context, from cognitive processing fluency, and from a learner’s affective states and self-concept (van Merrienboer and de Bruin, 2019). Metacognitive skills are required in *accurate monitoring* of progress, and in adapting strategies where necessary to support maintenance of effort (Panadero, 2017), and alignment of strategies to achieve goals (*adaptive control*). The ability to synthesize internal information and that from others in assessing one’s own work accurately is emphasized in *self-evaluative capacity* which also includes *reflexivity* in being able to effectively ‘step outside of oneself’ to objectively review lessons learnt and to make adaptations in one’s approach for the future.

In supporting SRAF development with academics we focused on high level metacognitive skillsets given that these skillsets are known to have the most impact on student learning outcomes (Dinsmore, 2017; Schneider and Preckel, 2017). This included firstly, a focus on *students’ self-efficacy and goal-setting* given the tendency for higher education students to have better results when interventions are aimed at motivational and emotional aspects of learning (Panadero, 2017; Van der Zanden et al., 2019). Efficacy beliefs are positively related to effective self-regulated learning (SRL) processes (Pintrich and Zusho, 2007) and according to Pintrich (2004), a much better predictor of performance than task value. Addressing goals and self-efficacy is thought to be especially impactful given the strong connections between goal orientation, control (academic self-efficacy) and affect, as explained in Pekrun’s control-value theory of achievement emotion (Pekrun et al., 2002, 2006).

Secondly, we looked at *metacognitive strategy instruction* in assessment and feedback which includes supporting students’ (i) understanding of strengths and weaknesses in relation to the demands of a task; (ii) strategy choice and effective use of strategy; (iii) internalization of standards in recognizing what good work is in supporting accuracy of monitoring and evaluation of work; (iv) recognition of feedback opportunities (cues) and developing effective feedback strategy use (processing and application skills); (iv) evaluation of the quality of approaches used, and in relation to accurate reading of context and task. This emphasis on SRL approaches combining metacognitive and motivational strategies is warranted given that they have the highest effects on student learning outcomes (Dignath et al., 2008). The importance of strategy instruction on student learning outcomes is established (Hattie et al., 1996; Schneider and Preckel, 2017). Metacognitive monitoring is essential in impacting outcomes (DiFrancesca et al., 2016). Donker et al. (2014) also found from meta-analytical research on 95 interventions that the effectiveness of strategy instruction on performance was enhanced when interventions included general metacognitive knowledge about when, why, how, and which strategy to use, taught students how to plan, and addressed task value.

In attending to motivational aspects of learning and acquisition of high level SRL skills, and taking account of information processing and socio-cognitive aspects of learning, we considered key features of assessment design and the environment that could support the development of students’ high level self-regulatory assessment and feedback skills drawing on EAT. SRL skills development takes place within specific contexts, and the extent to which the context enhances or reduces the potential impact of SRL strategy development on student performance is central to the EAT framework that we drew upon in this research.

### 3.3 The role of assessment design in supporting SRAF

The quality of assessment design impacts the efficacy of academics’ and students’ SRL skills development (Hawe and Dixon, 2017; Evans et al., 2021). It is important to address academics’ and students’ starting points, and their beliefs and conceptions about assessment in supporting SRAF (Evans and Waring, 2021). Essential elements of assessment design that support SRAF, drawing on EAT, Evans (2016, 2022), include: (i) engagement of students in working with academics to develop shared understandings of SRAF; (ii) embedding SRAF in all aspects of assessment design to support students’ progressive development of core knowledge, understanding, and skills; (iii) ensuring the balance and distribution of assessment activities is conducive to deep approaches to learning (e.g., positioning feedback so that it can be used to improve work), (iv) training students in what constitutes quality so they can gain an appreciation of quality for themselves; (v) supporting students as active agents of assessment and feedback change with clear roles and responsibilities, and opportunities to engage fully in all aspects of the assessment process as part of team ownership; (vi) understanding how a course as a whole is engineered, and how different assessment elements fit together (Bass, 2012), and (vii) ensuring learning outcomes are focused on student attainment of high level SRL skills (Brown et al., 2016). To create the conditions to support SRAF, cognitivist information processing, and socio-cognitivist perspectives on SRL were considered (See Table 1).

**Table 1:** Developing students’ self-regulatory skills within assessment design.

EAT	To what extent do students…	To what extent do academics….	SRL constructs supporting…..
AL1	Have an accurate understanding of the task and are able to set appropriate goals?	Make task requirements explicit? Engage students in developing assessment criteria? Provide opportunities to clarify what good is?	Task analysis Goal-setting Planning Meta-memory
AL2	Have a clear understanding of course requirements and the relationships between them?	Show how elements of the course fit together? Clarify the relationships between assessment tasks for the course?	Task discrimination and management Planning regulation of task
AL3	Have a clear understanding of their role and confidently engage with academics on assessment and feedback?	Make the student role in assessment explicit? Scaffold learning to build confidence and support students’ engagement in assessment?	Co-regulation with a more knowledgeable other (person /resource) Agentic engagement Self-efficacy
AL4	Have a clear understanding of what knowledge and skills are valued within the discipline and how to master them?	Signpost key knowledge and skills? Focus on student understanding of threshold concepts? Embed skills training throughout a course? Make what it is ‘*to think, act and be’* in a discipline explicit?	Contextual regulation Adaptive control
AF1	Distinguish what is valuable feedback, how to get it and apply it?	Provide training in how to recognize cues? Focus feedback on higher level concerns? Check student understanding of feedback?	Cue consciousness Information filtering Emotional regulation
AF2	Make best use of opportunities to test their understanding?	Provide early opportunities for students to test their understanding? Focus activities to learner needs?	Cue consciousness Self-monitoring and evaluation
AF3	Use peer learning opportunities effectively?	Train students in how to seek, give, and use peer feedback well? Ensure peer activities are authentic?	Shared regulation Relational skills -noticing
AF4	Accurately evaluate the quality of their work?	Embed self-assessment activities throughout the course?	Self-evaluation Reflexivity
AD1	Understand assessment regulations?	Engage students in marking and moderation?	Internalization of standards Monitoring accuracy
AD2	Know what a deep approach to learning is and how to realize it?	Ensure tasks require student ownership? Challenge students to adapt ideas and apply to new contexts?	Metacognitive skills in strategy selection and quality of use
AD3	Advocate effectively to ensure their needs are met?	Embed inclusivity in assessment design? Ensure accessibility of all resources? Reduce cognitive load?	Self-advocacy and self-determination skills Cognitive processing
AD4	Engage in developing assessment and feedback practice with academics?	Embed opportunities in courses for students to feedback on the quality of assessment design? Ensure co-creation embedded in design?	Critical reflection Agentic engagement

Supporting students’ accurate interpretation of tasks, requires an emphasis on making the requirements of the task explicit supported by clear signposting of information to reduce cognitive load (i.e., the amount of resource that a learner can devote to dealing with one task given the limits of working memory capacity) (Sweller et al., 2011), and providing early opportunities to address learners’ assessment conceptions and poor use of strategies (DiFrancesca et al., 2016). To support deep understanding of assessment requirements, students need frequent opportunities to discuss and interrogate the meaning of assessment tasks in order to come to a consensus as to what counts as quality. Understanding of students’ starting points and their previous experiences of success are important in tailoring SRL skills development (Douglas et al., 2016; Kim and Shakory, 2017).

In assisting students’ planning and goal-setting, emphasis should be placed on making the requirements of the task explicit, explaining the rationale of the task to support buy-in and shared goals, and exploring academics’ and students’ beliefs and conceptions about their roles in assessment. Academics working with students to agree goals that support their perceptions that a task is manageable and doable is important in relation to supporting student self-efficacy and agency in the assessment process. Autonomy supportive approaches where students are encouraged to question their understandings, where the “rules of the game are laid bare”, and where students are enabled a degree of ownership of the assessment process with academics are impactful (Panadero and Alonso-Tapia, 2013; Kumar et al., 2018; Evans, 2022).

In supporting students’ operationalization and effective monitoring and completion of assessment and feedback tasks, early opportunities to test their understanding, and explicit demonstration and modeling of effective strategies with them are important. Students need opportunities to practice, implement and evolve their metacognitive strategy use, and within relevant contexts (Zimmerman, 2000). Ensuring feedback is placed effectively to enable students the time to internalize and apply it, and ensuring feedback is focused on how students can enhance their skills development with examples of how to do so, are important. Similarly, facilitating students’ self-evaluation skills requires opportunities for students to test their understanding throughout their courses through being actively involved in activities which require them to exemplify their understandings (e.g., writing of practice and final tests; marking and moderation of work, constant comparison of work) to establish the merits and limitations of different approaches (Nicol, 2022). Eva and Regehr (2011, p. 327) argued the importance of creating situations for learners [and academics] to experience the limits of their competence in the presence of feedback with improvement strategies tailored to those experiences rather than self-assessment alone. Emphasis is therefore placed on supporting learners to assess their own strengths and weaknesses and to adapt their strategies according to task needs.

## 4 Aims

In working with academics, a key aim of our research was to support the translation of SRAF into practice in higher education through the following objectives as outlined below.

- Objective 1: To undertake a pilot study to clarify the factor structure of the SRAF scale.
- Objective 2: To ascertain academics’ perceived use of SRAF practice and professional development needs, and the relationship between use and needs.
- Objective 3: To explore the relevance of our findings for professional development of SRAF in higher education.

### 4.1 Development of the SRAF scale items

Research was undertaken with colleagues at four higher education institutions in Spain, Portugal, and the UK (two UK universities) to develop and implement a SRAF approach using the EAT framework (Evans, 2016, 2022). A multi-step methodological approach comprising the following elements was implemented:

- *Identification of SRL* var*iables that demonstrated maximum impact on student learning.* An extensive narrative review of the literature on SRL was undertaken to explore the relative effectiveness of self-regulation variables on student learning outcomes (Evans et al., 2021).
- *Emphasis was placed on high-level metacognitive self-regulatory skills* drawing on Dinsmore’s (2017) notions of conditional use (selection of appropriate strategies) and quality (using strategies well) aligned with Schneider and Preckel’s (2017) analysis which identified that the most successful students were those who were discerning in what they attended to in learning (Evans and Waring, 2021, 2023a). The interrelationships between metacognitive, cognitive, and affective dimensions of self-regulation in assessment and feedback were acknowledged (Vermunt and Verloop, 1999; Dinsmore, 2017).
- *Use of frameworks and tools to support understanding of SRAF*
  - EAT was used with academics to explore the self-regulatory skills needed to be successful in managing the requirements of assessment and feedback in all 12 sub-dimensions of EAT (Evans et al., 2022a; Evans, 2022).
  - *A SRL skills framework* evolved from EAT was used to support academics’ in thinking about the metacognitive skills required at each stage of a typical self-regulatory process (forethought, planning and goal-setting, performing a task and monitoring progress in relation to goals, and evaluating the extent to which goals had been met, and future actions) (Dinsmore, 2017; Seufert, 2018). Table 1 provides a summary of the SRL skills framework aligned with the sub-dimensions of EAT.
- Project leads and their teams in four institutions, as part of the wider project work on supporting SRAF skills development, engaged in two initial *core SRAF training sessions* each (eight in total) to explore approaches to using SRAF, with follow up work with project teams which provided important information on contextual affordances and barriers.
- Development of *reward and recognition frameworks* and online resources to support and recognize academics’ achievements (Evans et al., 2022b).

### 4.2 Participants

The online SRAF survey was distributed via project leads in the UK, Spain, and Portugal to academic colleagues in their institutions and their wider networks to ascertain the SRAF support academics’ wanted (support required), and perceived frequency of use of SRAF activities (practice frequency). This work is important given the lack of research exploring the gaps between academics’ knowledge of SRAF and implementation of it, and the need for robust measures to assist understanding of academics’ experiences of learning about and applying SRAF in practice.

Our initial sample size for analysis was *n* = 207. We removed observations from 4 participants who we considered to have submitted erroneous responses. Academics from 25 countries, including 115 higher education institutions contributed to this research. Most responses were from Portugal (*n* = 49, 24%), UK (*n* = 36, 18%), and Spain (*n* = 28, 13.7%) where lead partners were based. There were 103 (50.5%) males, 95 females (46.6%), and 6 academics (2.94%) not reporting their gender. Other key countries represented in the data included Greece (*n* = 29, 14.2%), and Brazil (*n* = 20, 9.8%), with the remaining 20% of respondents coming from individual associates of core partners from 20 countries. There was a broad distribution of respondents from across disciplines with 72 (35.3%) from STEM, 50 (25.5%) from medicine and related disciplines, 42 (20.6%) from social sciences, 35 (17.2%) from arts and humanities, and two colleagues whose roles were across disciplines. One hundred and eighteen academics (57.8%) identified their primary role was teaching, and 86 (42.16%) participants identified their main role was research. In relation to years of experience in higher education the profile of respondents was skewed toward those who had more experience in higher education. One hundred and thirty-three (65%) of respondents had 16 years or more, 27 (13.3%) had 11–15 years, 23 (11.3%) had 6–10 years, 15 (7.4%) had 2–5 years, and 6 (3%) had less than two years’ experience.

Ethical approval for the collection and use of data was obtained from the School Research Ethics Committee of the School of Biosciences, Cardiff University, UK, in accordance with institutional ethics policy and partner institutions ethical clearance arrangements and in relation to General Data Protection Requirements (GDPR). The purposes of the data collection were made clear to all potential respondents in line with ethical consent procedures, and all participants had the right to have their data withdrawn at any time.

### 4.3 The self-regulatory assessment and feedback scale

We were keen to identify participants’ perceptions of the support they required in developing SRAF, and against a marker of what SRAF practices they felt they currently used frequently in their practice.

All participants were asked to complete two versions of the questionnaire scale, one asking academics what support they required in SRAF (SR), and the other asking them about their perceived practice frequency of SRAF (PF). Participants were asked to score items on a five point likert scale. For example, for SR (personal needs for training: 0 = not needed, 1 = very low to 5 = most needed) and for PF (frequency of use of SRAF approaches: 0 = not used, 1 = used very rarely to 5 = used very often).

The SRAF scale comprised 21 items generated from the EAT Framework and research on high level self-regulatory skills (Dinsmore, 2017; Evans and Waring, 2021). The questions highlight the importance of addressing cognitive, affective, and metacognitive aspects of self-regulation. For example, (i) clarifying how assessment elements fit together and facilitating student access to concepts by making core concepts explicit, thereby reducing cognitive load (cognitive); (ii) explaining the rationale underpinning assessment design, and the role of the student in assessment and feedback (affective), and (iii) ensuring opportunities for students to test their understanding through repeated opportunities to engage actively in assessment processes so as to support internalization of learning processes (Sadler, 2009; Nicol, 2022) (metacognitive aspects).

In developing the SRAF scale items consideration was also given to the metacognitive skills needed at each stage of the self-regulatory process (Pintrich, 2004) to include planning and goal-setting, including activating perceptions of a task, and one’s role in it, utilizing strategies to complete the task including ongoing monitoring of progress, and evaluation of the extent to which goals have been met. Self-regulatory assessment practices targeted included academics’ support of students’ (i) planning and goal setting, (ii) self-efficacy, (iii) internalization of standards, (iv) dispositions in encouraging a mastery approach to learning, (v) ability to adapt and transfer learning to new contexts, (vi) management of feedback, (vii) metacognitive skills regarding their self-awareness of their strengths and weaknesses, and (viii) ability to accurately judge the quality of their own work.

In working with academics we explored the high level self-regulatory skills required to support effective assessment and feedback and how these could be applied in different cultural contexts (Table 1) drawing on the 12 sub-dimensions of EAT (Evans, 2022). Importantly, we intentionally focused on participatory approaches in how academics work *with* students in partnership to support SRL development. Participants’ responses to the 21 items comprising the scale in relation to support required are depicted in Table 2.

**Table 2:** Descriptive statistics for 21 original items of SRAF (SR) scale items.

Related constructs		SRAF items	M	SD	Skewness	Kurtosis
Prior knowledge	1	I review data on students’ starting points and regularly review their progress on assessment tasks to check what it is working well or not, and for whom.	3.0	1.18	−0.66	0.1
Meta/cognitive strategies	2	I explain how the assessment tasks in the course I am teaching on relate to other courses students are taking as part of their program.	2.73	1.34	−0.26	−0.85
Cognitive (Cues)	3	I signpost the key skills students need to learn in their course.	2.69	1.30	−0.22	−0.81
Cognitive (Load)	4	I carefully consider how I introduce new ideas to students so as to not overload them with too much complex information at one point.	2.75	1.29	−0.14	−0.90
Internalization of standards	5	I embed self-assessment activities throughout a course so students get opportunities to test their levels of understanding for themselves.	3.16	1.19	−0.60	−0.35
Feedback utilization	6	I time feedback opportunities carefully so that they have maximum impact in supporting students’ development of knowledge and skills for future work.	3.0	1.21	−0.61	−0.33
Affective (motivation) and cognitive	7	I explain the rationale underpinning the design of assessment with students.	2.73	1.35	−0.36	−0.77
Shared regulation	8	I design assessments that reward students’ ability to work collaboratively to achieve shared goals.	3.11	1.17	−0.65	−0.19
Agency and autonomy	9	I encourage students to take responsibility for their own learning.	3.06	1.45	−0.45	−0.8
	10	I actively involve students during the course in providing feedback on the quality of learning activities.	3.10	1.31	−0.55	−0.41
Planning and Goals	11	I place emphasis on supporting students’ planning skills (how they identify the requirements of a task and plan for managing the successful completion of it).	3.04	1.16	−0.67	0.18
	12	I work with students to help them identify and agree goals for their learning.	3.02	1.20	−0.43	−0.35
Self-efficacy	13	I explore with students their beliefs in their ability to do well and how they can enhance their confidence in their learning.	3.09	1.20	−0.62	−0.21
Internalization of standards	14	I engage students in developing marking criteria for assessments.	3.02	1.25	−0.57	−0.35
	15	I work with students to help them understand the marking criteria for assessments.	2.87	1.30	−0.43	−0.50
Deep approach	16	I encourage students to explore the meaning behind ideas for themselves, and to think about how they can apply what they have learnt to create new understandings.	2.99	1.30	−0.48	−0.48
Feedback regulation	17	I provide guidance to students on how to recognize and seek different sources of feedback, and to use feedback effectively to enhance performance on subsequent tasks.	3.14	1.26	−0.57	−0.17
Shared and co-regulation – relational skills	18	I train students in how to work effectively together and to support each other’s learning.	3.09	1.22	−0.51	−0.31
Metacognition reflexivity	19	I work with students to enable them to have a better understanding of what their strengths and weaknesses are in relation to the core knowledge and skills required in the course, and how to address these.	3.18	1.17	−0.49	0.01
	20	I share data with students so that they can see how certain approaches to learning may be more effective than others.	3.10	1.27	−0.56	−0.34
Self-evaluative judgment	21	I work with students to develop their monitoring and evaluation skills so that they are able to accurately critically appraise how well they are doing.	3.19	1.18	−0.53	−0.14

## 5 Data analysis

### 5.1 Establishing the factor structure of the SRAF questionnaire

All data analyses were conducted using R (version 4.3.1, R Core Team, 2023). The SRAF scale comprised 21-items.

The SRAF scale survey was distributed online to academics who were asked about which of the 21 items they most wanted professional development support in, and which of the 21 items they perceived they used most frequently in their teaching.

We anticipated that factors arising from the underlying concepts would be correlated, hence we performed iterative exploratory factor analysis (EFA) with oblique (Promax) rotation. The EFA was undertaken to evaluate the dimensional structure and internal consistency of the SRAF scale, and reliability analysis was undertaken for academics’ perceptions of support required (SR) and practice frequency (PF). We explored mean absolute difference (MAD) to compare the differences in academics’ responses to the items on the support required (SR) and practice frequency (PF).

First, we undertook initial data screening of our 21 items. Following Evans and Zhu (2023), we considered items for elimination if (i) absolute skew values were >2.0 and absolute kurtosis values were <7.0 (Kim, 2013), (ii) items had a low average inter-item correlation ( <0.3 ), (iii) items had very high average inter-item correlation suggesting multicollinearity ( >0.9 ), and (iv) a low inter-total correlation ( <0.3 ).

Descriptive statistics for each item were calculated using the *descriptives* function from the *psych* package (Revelle, 2023). The MAD was calculated by taking the mean of the absolute difference between PF and SR for each respondent. Inter-item correlations were calculated using the *corrr* package (Kuhn et al., 2022); and item-total correlations were calculated using the *performance* package (Lüdecke et al., 2021).

We deemed items suitable for EFA if (i) Kaiser-Meyer-Olkin (KMO) factor adequacy score was >0.7 (Kaiser, 1974), (ii) a Bartlett’s test of sphericity returned a significant result ( p<0.05 ), and (iii) the determinant of the correlation matrix was >0.00001 (Yong and Pearce, 2013). To determine the maximum number of factors to explore in each EFA, we considered multiple sources of evidence: (i) the number of eigenvalues exceeding Kaiser’s criterion ( >1 ) (Kaiser, 1974), (ii) visual inspection of scree plots (Ledesma et al., 2015), (iii) parallel analysis using both principal components and common factor analysis extraction methods (Hayton et al., 2004), (iv) Minimum Average Partial (MAP) tests (Velicer, 1976), sequential chi-square model tests (Auerswald and Moshagen, 2019) and empirical Kaiser criterion scores (Braeken and van Assen, 2017).

We examined EFA results in the context of the percentage cumulative variance explained (Costello and Osborne, 2005), and according to factor loadings. Specifically, we eliminated items with factor loadings <0.45 (Hair et al., 2010) or with cross-loadings on two or more factors without a difference of >0.3 . Following EFA, reliability analysis was conducted to determine internal consistency of items loading onto their associated factors. This was done using Cronbach’s Alpha ( α ) with items deemed reliable if α>0.7 (Taber, 2018).

## 6 Results

### 6.1 Confirming the factors underpinning the SRAF scale

Descriptive statistics for individual items of the SRAF survey for Support Required (SR) are presented in Table 2. The 21 SRAF scale items entered the first EFA using data from 173 participants; there were 30 missing cases. EFA resulted in elimination of four items (items 1, 10, 15, 16) due to low loadings <0.45; two items (5 and 14) were borderline, and a decision was made to retain these.

The final scale with the remaining 17 items yielded a Kaiser MSA value of 0.93 and a significant Bartlett’s test of sphericity (*χ*^2^ (136) = 2102.00, *p* < 0.001). These results verified that the SRAF (SR) sample was suitable for factor analysis. Two components had eigenvalues over 1. Inspection of the scree plot further recommended a two-factor solution.

The final two-factor solution (shown in Table 3) accounted for 57% of the overall variance with 95% reliability: factor 1 containing nine items (31%) and factor 2 containing eight items (26%). Internal reliabilities for the two factors suggest each subscale as a reliable measure (*α* = 0.94 for factor one with item-total correlation ranging from 0.53 to 0.65; and *α* = 0.0.9 for factor two, with item-total correlation ranging from 0.49 to 0.59). From review of the loadings on the two factors, factor 1 represented *Supporting Students’ SRAF Skills Development,* and factor two represented *Creating the Conditions for SRAF.* According to the component correlation matrix there was a strong positive correlation between the two factors (*r* = 0.75) which was expected given the highly interconnected nature of the constructs we were exploring. The same two factor solution was verified in running iterative ERA on academics’ responses to practice frequency items with two items having eigenvalues over 1. However, four questionnaire items (questions 2, 9, 11, 18) were removed as the loadings were below 0.45 for the practice frequency questions.

**Table 3:** SRAF (SR) summary of exploratory factor analysis with Cronbach’s alpha (*n* = 173).

Rotated factor loadings		
SRAF (SR) items	Factor 1	Factor 2
2		0.74
3		0.85
4		0.9
5		0.5
6		0.69
7		0.82
8		0.54
9		0.57
11	0.67	
12	0.64	
13	0.71	
14	0.52	
17	0.65	
18	0.77	
19	0.97	
20	0.74	
21	0.97	
% of variance explained	31%	26%
Internal reliability (Cronbach’s *a*)	0.94	0.90

Item loadings < 0.45 are suppressed.

### 6.2 Academics’ perceptions of support required in developing SRAF approaches

The means and standard deviations for the 17 items comprising the SRAF Support Required (SR) scale are provided in Table 2. Overall, academics wanted most support with students’ metacognitive strategy development, and least support with assessment literacy and supporting students’ cognitive skills (e.g., managing cognitive load). Academics most wanted assistance with supporting students’ monitoring and evaluation skills, their self-awareness of strengths and weaknesses in relation to course demands. In *Creating the Conditions for SRAF* academics most wanted support with how to embed self-assessment within assessment design.

### 6.3 The relationship between support required and reported frequency of use of SRAF

Using the approach used by Dinsmore et al. (2024), we calculated the mean absolute difference (MAD) to explore the gap between the SRAF approaches academics reported wanting most support with, and those they reported using most (Table 4). A larger MAD represented a bigger discrepancy between the score for practice frequency and support required. MAD was calculated for the 13 items that loaded above 0.45 on both sets of SRAF questions (practice frequency (PF) and support required (SR)).

**Table 4:** Mean Absolute Difference between responses to frequency of practice and support required.

F	SRAF ITEMS	Practice Frequency		Support required		MAD	SD	MAD	Category
	Factor	M	Rank	M	Rank	M		Rank	
F2	**Factor 2**								
1	Reviewing students starting points								
2	Showing how assessment tasks link together			2.73	15				
3	Signposting key skills	3.72	2	2.69	17	1.47	1.32	3	PF High SR Low
4	Managing cognitive load	3.99	1	2.75	14	1.49	1.39	2	PF High SR Low
5	Embedding self-assessment activities	3.17	10	3.16	3	1.26	1.3	8	PF Low SR High
6	Placing feedback to maximize impacts	3.39	4	3.00	13	1.16	1.17	12	PF High SR Low
7	Explaining the rationale underpinning assessment	3.65	3	2.73	15	1.52	1.37	1	PF High SR Low
8	Rewarding collaborative work to support shared goals	3.36	5	3.10	5	1.14	1.08	13	PF High SR High
9	Student responsibility for learning			3.06	9				
10	Gaining student feedback on the quality of learning activities								
F1	**Factor 1**								
11	Emphasis on planning skills, task recognition, and strategy			3.04	10				
12	Agreeing goals for learning	3.11	11	3.02	11	1.28	1.24	6	PF Low SR High
13	Academic self-efficacy	3.26	7	3.09	7	1.23	1.16	10	PF Low SR High
14	Student engagement in developing marking criteria	2.43	13	3.02	11	1.28	1.24	6	PF Low SR High
15	Helping students to understand criteria								
16	Encouraging a deep approach								
17	Feedback guidance	3.35	6	3.14	4	1.38	1.29	5	PF High SR High
18	Training students to work collaboratively			3.09	7				
19	Metacognitive strategy	3.22	8	3.18	2	1.23	1.14	10	PF Low SR High
20	Using data with students	3.21	9	3.10	5	1.44	1.32	4	PF Low SR High
21	Monitoring and evaluation	3.01	12	3.19	1	1.24	1.19	9	PF Low SR High

With the exception of item ten (embedding self-assessment within assessment design), academics’ reported giving most attention to items comprising *Creating the Conditions for SRAF* with emphasis on supporting students’ assessment literacy which is reflective on the emphasis there has been on this in research for last ten years (Zhu and Evans, 2022). Academics reported greatest focus on supporting students’ cognitive skills (e.g., access to assessment and feedback by reducing cognitive load, signposting key skills, and explaining the rationale underpinning assessment). From an assessment design perspective, academics’ focused efforts on the placement of feedback to maximize support for student learning and on rewarding collaborative practices. An emphasis on *Supporting Students’ SRAF Skills Developmen*t was less evident, with academics’ reporting least attention being placed on engaging students in developing assessment criteria, supporting monitoring and evaluation skills, and agreeing goals for learning.

**Figure 2:**
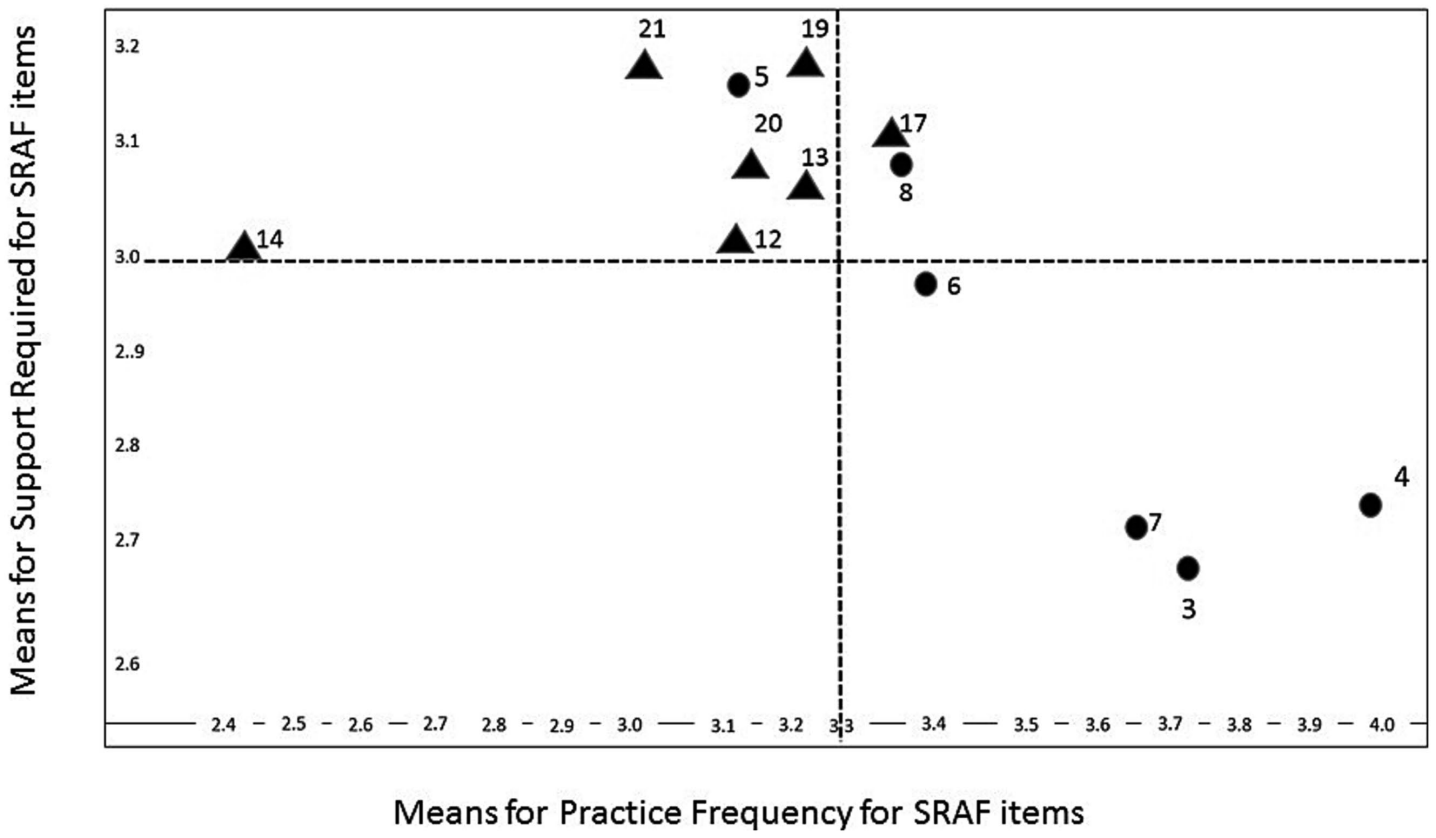
The relationship between practice frequency and support required for SRAF items.

Figure 2 illustrates the relationship between PF and SR for each item of SRAF that could be compared across the two SRAF surveys (13 items in total). Three clusters were identified from the data from a possible four combinations (Evans and Waring, 2023a).

- Cluster 1: high use (PF) and high interest (SR) (items 17, 8)
- Cluster 2: low use (PF) and high interest (SR) (items, 5, 12, 13, 14, 19, 20, 21)
- Cluster 3: low use (PF) and low interest (SR) (no items)
- Cluster 4: high use (PF) and low interest (SR) (3, 4, 6, 7)

Perceived high usage and high interest items (Cluster 1) relate to supporting students’ feedback skills, and abilities to work collaboratively in developing co- and shared regulatory practices with peers. High usage and low interest items (Cluster 4) relate to areas that academics feel are embedded in their practice and do not need more support with; these include, for example, supporting students’ cognitive access to assessment and aspects of assessment design to ensure that feedback feeds forward. Low usage and high interest items (Cluster 2) form the largest group in our findings and are largely focused on academics’ metacognitive skills development and enabling the embedding of such within assessment design. These items include the need to support students’ goal-setting, engagement in co-creation of assessment criteria; use of data with students, and monitoring and evaluation skills; areas reported as less frequently used in practice by academics.

In reviewing missing items (those that did not load at sufficient levels on either factor), Item 1 ‘*Awareness of students’ starting points, and ongoing review of progress*’ is an area that is known to be very important in self-regulated learning in impacting the effectiveness of instructional techniques (Fyfe and Rittle Johnson, 2016), however, academics’ reported relatively low usage of, and low interest in getting more support in this area. Evans and Waring (2023a) highlight the importance of using SRAF tools with colleagues to surface the relevance of key constructs and to demonstrate how to integrate SRAF into practice in a manageable way that is relevant to context. Academics in training sessions integral to this research identified the main reason for not using certain SRAF approaches was due to not knowing about them in the first place, providing face validity for Evans and Waring’s cluster 3 category.

## 7 Discussion

### 7.1 Confirming reliability, validity, and underpinning SRAF constructs

Construct validity was established through exploratory factor analysis (EFA). Our end product included a two-factor scale. Content validity and internal consistency reliability supported evidence of construct validity which was also supported by the theoretical underpinnings of the SRAF scales.

Two constructs *Supporting Students’ SRAF Skills Development* and *Creating Conditions for SRAF* were established. Academics’ placed most emphasis on supporting students’ metacognitive skills development, and less attention on motivational aspects such as goal-setting, and planning aspects of self-regulation. This finding was congruent with academics’ reported focus on *Creating the Conditions for SRAF* with emphasis on cognitive skills development. Our findings, in many respects, are similar to those of Dinsmore et al. (2024) in that academics reported greater use of cognitive strategies compared to metacognitive and motivational dimensions of learning. Identifying that these themes are common across very different samples suggests the potential generalizability of these findings which would need further verification across wider contexts. These results are not surprising given that research suggests less emphasis is being placed on developing students’ goal-setting strategies compared to other aspects of self-regulation (e.g., feedback-using skills) in higher education (Evans and Waring, 2021, 2023a,b). This finding is congruent with evidence suggesting that much emphasis has been placed on reflection on feedback on performance on a task at the expense of time spent on supporting students’ planning and goal setting in assessment and feedback (Farrell et al., 2017). This matters because of the importance of planning skills and goal development in impacting student outcomes, and suggests an important gap between practice and research that needs to be addressed (Panadero, 2017).

Similarly, academics who reported high usage of items loaded on *Creating Conditions for SRAF* were likely to report lower usage on *Supporting Students’ SRAF Skills Development.* To be most effective SRAF requires both aspects of SRAF to work in unison (Evans, 2013). The findings may reflect the stage of professional development that academics are at, in that staff identified the need for support in facilitating SRAF skills development with students.

### 7.2 Strengths and areas for development

#### 7.2.1 Scale considerations

One of the greatest strengths of the SRAF scale was in its practical use as a powerful heuristic to guide discussions on effective SRAF with academics. A common criticism of self-rating scales is the degree of discrepancy between actual and perceived behaviors (Chrystal et al., 2019; Uher, 2023). The SRAF scale focuses on perceived training needs and estimates of frequency of use of SRAF. The scale was useful in supporting academics to identify high level SRL skills and in showing them how to implement SRAF using EAT. It provided a valuable mechanism to support discussions about what effective SRAF practice looked like in different contexts. A core aspect of building SRAF competency is in unpacking conceptions and beliefs about what constitutes good practice and why.

In scrutinizing the properties of the SRAF scale it demonstrated strong internal reliability. The sample size was adequate for preliminary EFA but needed a larger sample in order to perform second stage confirmatory factor analysis. This initial pilot was valuable in identifying the strength of the scale but also indicated areas where it could be further refined.

While the same two underpinning factors were identified in questions focusing on the support academics’ wanted and their perception of practice frequency of SRAF, the discrepancy between the number of items that loaded on questions about support and those that loaded on frequency of use of SRAF was a concern. Some of the pilot SRAF items that would have been expected to load on the two identified dimensions did not have loadings above 0.45, which was our cut off point for further EFA, suggesting the need for further refinement of the items comprising the scale. In looking at academics’ scores on some of these excluded items it is interesting to note that item one about reviewing data on students’ starting points and ongoing checking of progress is integral to developing an inclusive culture to support SRAF. Effective use of data to enhance assessment design is a significant issue for higher education (Evans et al., 2019). Item 15 on working *with* students to support their understanding of assessment criteria is fundamental to students being clear about the expectations of assessment. Item 16 on supporting students’ development of a deep approach to learning should be central to assessment design but it is a complex construct. Traditionally a deep approach is associated with the intention to understand, but it also requires understanding of the process of learning within specific contexts (McCune and Entwistle, 2011), and discernment in knowing what the most appropriate strategies are to master a task (Evans and Vermunt, 2013). This construct needs further unpacking as it has many constituent parts. The complexity inherent in individual self-regulatory constructs is a key challenge in SRL skills research (Carless and Boud, 2019). For example, in looking at evaluative judgment, Luo et al. (2023) have identified five constructs involved including understanding of the context (i.e., assessment literacy) and the interplay of metacognitive, affective, and cognitive components which aligns with EAT (Evans, 2016, 2022). The key challenge is distilling the essential items that can best support academics’ understanding of key factors at play within SRAF, and across contexts.

Given the complexities of self-regulatory constructs and the need to develop clear understandings of them, and within discipline contexts (Evans and Waring, 2023a), there is a need to refine these items to explore different facets of them. The SRAF (SR) scale inter-item correlations suggest especially for factor 1, *Supporting Students’ SRAF Skills Developmen*t, that the scale could be enhanced to capture a broader bandwith of the construct (Piedmont, 2014).

Initial findings from this preliminary study are positive given alignment with comparable studies and testing of ideas with colleagues from very different cultural contexts (institution, country, discipline). Further work is needed to refine and test items with a larger sample that will permit further testing of the SRAF scale’s properties through exploratory and confirmatory (CFA) factor analyses as to its suitability for use with different samples. Our results to date are promising in this respect, given the similar findings in Dinsmore et al. (2024) when focusing on skills development (factor 1). In working collaboratively with academics and students it is possible to verify individual and team perceptions of strengths in areas of SRAF practice through peer feedback and open dialog around understanding of concepts, and evidence of effectiveness of SRAF approaches.

Subject to satisfactory CFA results, convergent validity can be explored through utilization of aligned frameworks and tools:

- A relationship between Dinsmore et al.’s (2024) self-regulatory assessment scale and dimension 1 of SRAF: *Supporting Students’ SRAF Skills Development* would be expected as they are both measuring self-regulatory skills use. Key differences are that Dinsmore et al. place greater emphasis on task value, whereas the SRAF scale, drawing on the EAT framework, places greater emphasis on partnership with students in supporting different phases of the self-regulatory cycle aligned to very specific SRAF practices, whereas Dinmore et al.’s scale emphasizes broader metacognitive skills.
- A relationship would be expected between the assessment engagement scale (*AES*) of Evans and Zhu (2023), and *Creating the Conditions to Support SRAF* (factor 2) given that the AES is focused on the extent to which assessment design supports SRAF, suggesting there should be strong alignment.
- Predictive validity can be explored through academics’ perceived engagement in SRAF, perceived self-efficacy in ability to implement SRAF, in impacting the quality of assessment design, and the extent to which students’ perceive that assessment design enables them to engage in SRAF (using the Assessment Engagement Scale (student version), Evans and Zhu, 2023).

#### 7.2.2 Wider methodological strengths and limitations

In developing the SRAF scale, a key strength of our sample was that it was representative of the higher education academic community in that it comprised international academics from a wide range of disciplines, research and teaching roles, and was well balanced with respect to gender. However, the breadth of the sample limited certain types of analyses at the individual institution level.

The testing of SRAF concepts with colleagues across different cultural contexts was effective in maximizing the utility and relevance of SRAF tools for an international audience, supporting translation of ideas into practice.

Focusing attention on academics’ perceptions of their use of SRAF and the professional development they wanted in SRAF was powerful in supporting the reframing of professional development activities to focus on key SRAF knowledge and skills gaps in specific contexts. Our research draws attention to the importance of exploring how academics assess the quality of their SRAF practice, and what evidence informs this process. A key question arising from this research was how those leading SRAF training are supported in bridging SRAF knowledge and practice gaps. In this article, our focus was purely on academic’s perceptions of this process. Further work is recommended on the perceptions of professional development staff in relation to how they perceive affordances and barriers in supporting the quality of SRAF professional development training aligned with the SRAF skillsets required within specific disciplines.

### 7.3 Implications of academics’ reported use of, and interest in, SRAF in supporting the professional development of SRAF

Academics reported greater use of cognitive strategies compared to metacognitive ones as also identified by Dinsmore et al. (2024) in a very different context. In contrast to Dinsmore et al. our sample of academics demonstrated high interest in learning more about how to support students’ goal-setting and academic self-efficacy. This finding may be related to the fact that our sample of academics was purposeful in that they were engaged in networks where we had been promoting the importance of attending to affective and motivational dimensions of self-regulation to include self-efficacy, goal-setting, and planning.

SRAF requires discernment in knowing which strategies to use in any given context, and how to use them well (Dinsmore, 2017); this is especially pertinent to SRAF professional development in higher education (Evans and Waring, 2023a). Dinsmore et al. (2024) argue that the nature of strategies used to support academics’ professional development in SRAF is dependent on academics’ use and interest in SRAF. Expectancy value theory is relevant to Dinsmore et al.’s argument (Wigfield and Eccles, 2000), in that to invest in SRAF training, academics need to have a reasonable expectation that such training will benefit them and their students.

Evans and Waring (2023a) argue that valuing of a task is insufficient in itself to gain engagement of academics in SRAF, drawing on the role of control value theory of achievement emotions in this (Pekrun et al., 2006). In supporting academics’ engagement in SRAF they highlight the importance of academics’ perceptions of competency (e.g., expectancy of successful outcomes, productive relationships with students), and support from others (colleagues, department, institution). The interaction of these variables impacts academics’ choice of metacognitive, cognitive, and affective strategies (e.g., help-seeking and managing one’s environment), with impacts on performance, satisfaction, and motivations, which also affect emotions and perceptions of competency, task value and goal orientation (Evans and Waring, 2021, p. 462).

**Figure 3:**
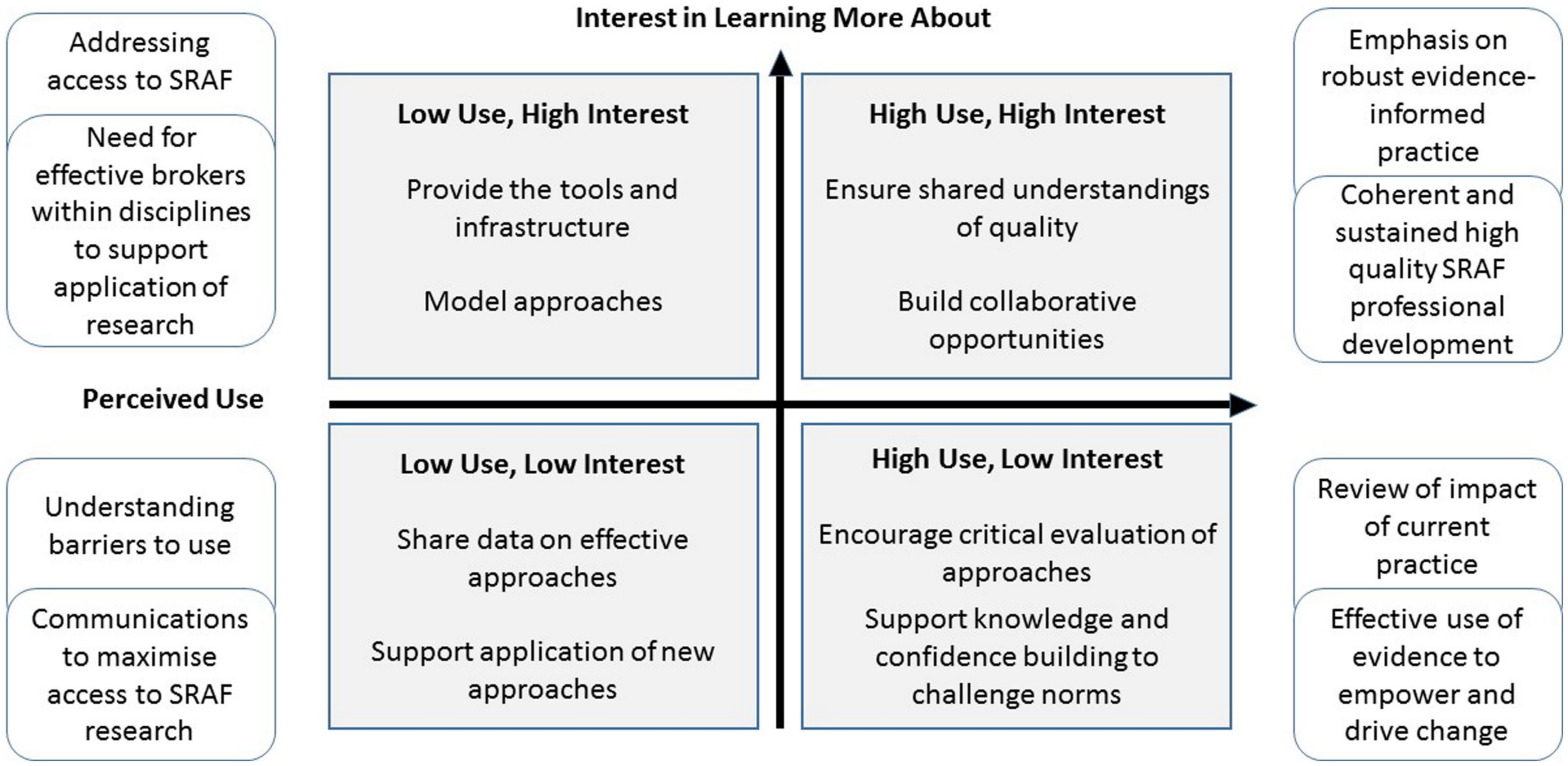
The perceived use of, and interest in developing, self-regulatory assessment and feedback skillsets, and associated challenges Adapted from Evans and Waring (2023a).

Figure 3, adapted from Evans and Waring (2023a) and drawing on Dinsmore et al., highlights that professional development strategies should take account of academics’ perceived use and interest in learning more about SRAF. There are a range of challenges in managing SRAF professional development dependent, for example, on academics’ dispositions, interests, and the contexts in which they work. For example, colleagues may report high usage of a particular strategy but ensuring shared understanding of what constitutes quality is difficult to achieve without an ongoing, co-ordinated and high quality SRAF professional development offer; an area in great need of attention in higher education (Ruiz and Panadero, 2023). Alternatively, academics may report high use of SRAF practices and little need for further development in them. Challenging ingrained positions on practice is difficult and requires a strong evidence-based approach to convince and empower individuals to make changes to established ways of working. Alternatively where low usage of SRAF is reported, barriers to access need to be addressed and the importance of brokers within disciplines to support change is imperative. Academics involved in SRAF networking activities highlighted that low use of SRAF was often related to lack of awareness of it and the strategies to support implementation of SRAF (Evans et al., 2019; Evans et al., 2021). In this respect we argue the importance of a coherent institution-wide communications strategy that supports networking and sustainability of SRAF through dissemination of effective research strategies to evaluate the relative value of using SRAF approaches to build a sustainable research and practice SRAF community.

**Figure 4:**
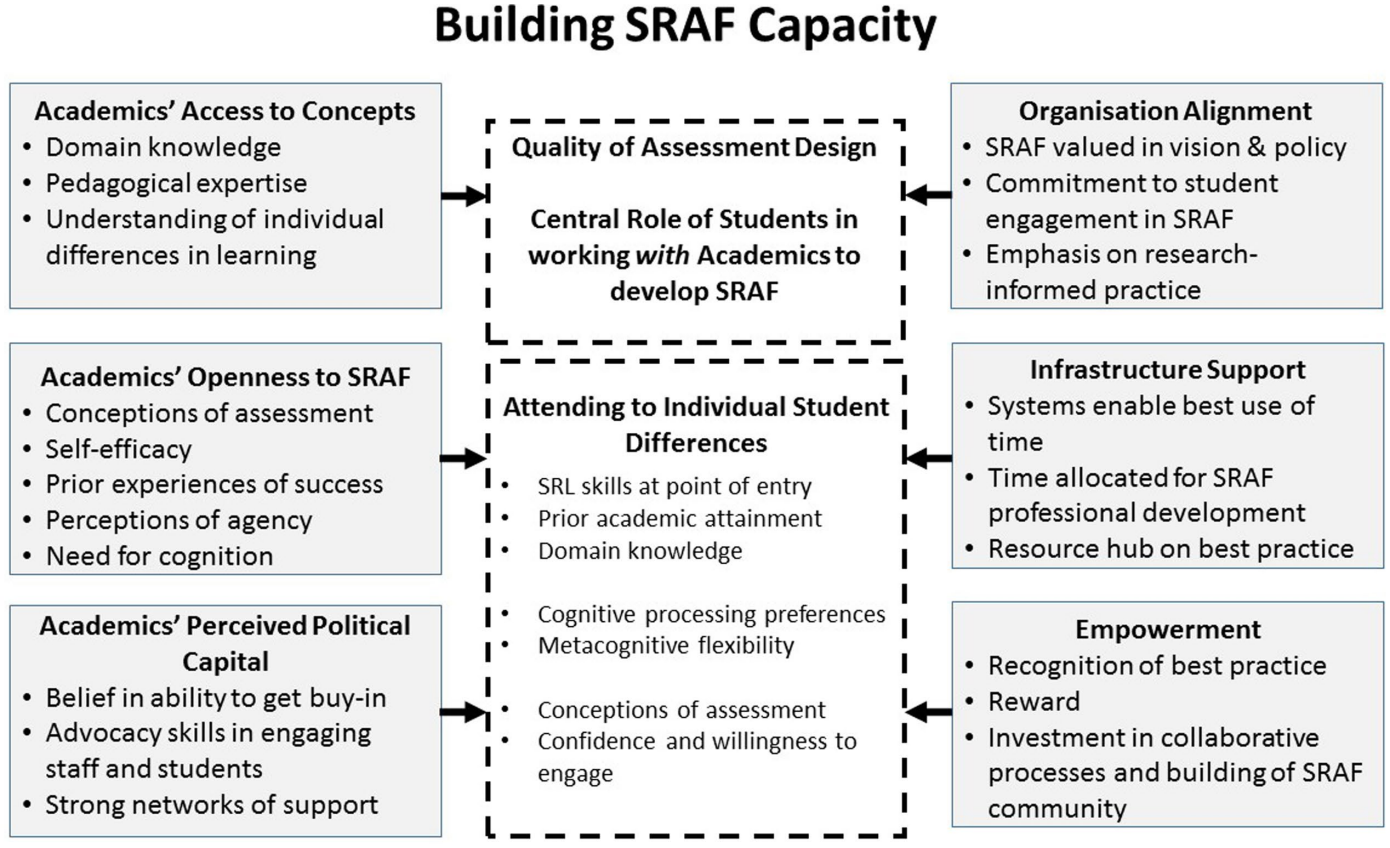
Building SRAF capacity (Evans and Waring, 2023b).

Through working in practice with academics and using the exploratory tools described in this article, it was possible to identify key challenges impacting use of SRAF; these align closely with those found in previous studies (Evans et al., 2019, 2021). In Figure 4, these factors are grouped into individual and organizational factors that work in unison to impact knowledge of, engagement in, and successful application of SRAF. The individual factors closely align with Winne’s (1996) identification of five key factors implicated in self-regulated learning (i.e., global dispositions, domain knowledge, knowledge of tactics and strategies, performance and regulation of tactics and strategies).

An emphasis is needed on academics’ self-regulation of assessment and feedback if they are to be best supported in developing these practices with their students (Russell et al., 2022). Using SRAF requires both a cognitive shift in academics’ understanding of how best to support students’ acquisition of SRL skills within a specific domain (Simper, 2020) and a seismic cultural shift in reconceptualizing assessment as a participatory process with a different role for the students in this process (Faherty, 2015). Simper (2020) aligns academics’ changes in thinking about assessment with the notion of threshold concepts (Meyer and Land, 2003). Changing one’s thinking about assessment is a challenging process which often involves changes in one’s ontological positioning (Land et al., 2005), which may also be in conflict with other colleagues’ views on assessment and the institution view of assessment. SRAF requires academics and students to reposition themselves and their roles in relation to each other (Chan and Chen, 2023), where assessment is no longer “done unto students”, and where students, while not ultimate authorities in assessment (Cook-Sather, 2014), have valid input into assessment (Evans, 2013, 2016). The challenge for academics is in supporting students to take on a number of different roles in assessment (input into assessment design, feedback, and marking), which requires developing focused training for students in how to take on these roles, and to understand the specific requirements associated with different types of roles.

The challenges impacting academics’ development and use of SRAF in practice drawing on information processing and socio-cultural perspectives are highlighted in Figure 4. At the individual level, in supporting SRAF three core areas from our work with academics have been identified relating to access to concepts, openness to new approaches, and perceived political capital in leveraging SRAF with their peers and their students (Evans et al., 2019). We argue that SRAF training needs to attend to these different areas, and to explore the relationships between them. While understanding of individual differences in learning could be encapsulated within pedagogical expertise given the need to focus training on this area, we have created a separate category for it.

Myyry et al. (2022) highlight the importance of addressing teacher self-efficacy. Academics’ perceptions of their agency and advocacy in leveraging change were key factors mentioned in this research in discussions around challenges in implementing SRAF. Academics mentioned difficulties in accessing SRAF concepts that were totally new to them in many cases, and needing access to the language and theoretical framing underpinning concepts and help in seeing how these ideas could be applied within their discipline. The EAT framework was useful in providing a concrete routemap of how to apply SRAF to practice, and through explicit labeling of key self-regulatory processes implicated in assessment (Table 1).

Figure 4 highlights the importance of institutional alignment in supporting academics’ implementation of SRAF through policy and strategy emphasizing students’ meaningful engagement in assessment underpinned by evidence-informed practice. A coherent, integrated and sustainable assessment strategy must take account of the roles of academics, professional services, technical support teams, wider stakeholders and students in assessment and feedback activities. The importance of effective infrastructure that takes away the “heavy lifting” of assessment (e.g., through automation of basic functions, agile policy to enable dynamic change in assessment, efficient marking and moderation systems) is emphasized. Prioritizing time for academics to work on SRAF embedded assessment designs and resources is seen as essential to ensure aligned assessment processes focused on supporting the progressive development of students’ self-regulatory skills. Empowering academics through recognizing and rewarding SRAF, and in supporting the building of collaborative communities that enable the sustained development of effective SRAF are important (Evans et al., 2022a,b). Integral to Figure 4 and central to it, is attending to students’ engagement in SRAF which parallels key constructs identified as central for academics (e.g., domain knowledge, processing styles, conceptions of assessment and confidence and willingness to engage). Greater understanding of the attributes that students bring into higher education is essential to complete the SRAF learning cycle.

## 8 Conclusions: implications for evolving SRAF research and practice

This article makes an important contribution to advancing assessment and feedback practice in higher education by highlighting the importance of supporting academics’ SRAF development if they are to effectively facilitate their students’ SRL skills development. This focus on SRAF is essential in supporting students’ learning in higher education. In bridging the research-practice divide, this article outlines conceptual and practical frameworks and tools to support the translation of SRAF concepts into practice. A considered and research-informed approach to academics’ professional development in SRAF is advocated to support academics in evaluating their practice, and in enabling focused attention on what matters in assessment and feedback as part of a self-regulatory approach.

In advancing understanding of SRAF, we identified two factors underpinning the SRAF scale: *Supporting Students’ SRAF Skills Development* and *Creating the Conditions for SRAF*. The strong internal reliability of the scales supported its use with academics although further work is needed to fully capture the high level SRL skills we were focusing on given the complexity of the SRL construct, and the need to test the scale items on larger samples. In this pilot study we focused on those self-regulatory behaviors known to have greatest impact on learning. Data captured from academics through this initial data gathering stage will be used to refine the scale items to capture greater breadth of SRAF.

One of the greatest benefits of the SRAF scale was in its use as a practical learning tool; a heuristic to guide academics in exploring high level SRL skills with their students. The SRAF scale was a valuable measure to explore academics’ views on the areas in which they perceived they needed most help in developing SRAF, and in comparison to their reported frequency of use of SRAF. The conceptual and practical tools developed to support SRAF implementation were powerful in raising awareness of the importance of developing students’ metacognitive skills, as evidenced in academics’ preference for training in this area, and in promoting a shift to a more evidence-informed approach to supporting students’ SRAF. Our findings highlight the importance of effective dissemination of information about core SRAF practices, and how to implement them in practice.

Further work is needed to better understand the processes involved in supporting academics’ understanding and use of SRAF. Figure 4 highlights a range of factors impacting academics’ use of SRAF which need consideration in the design of professional development to support academics’ understanding of SRAF.

A key challenge in supporting SRAF research and practice in higher education is in the complex interplay between the numerous self-regulatory concepts and processes involved in interaction with individuals and the contexts in which they are working. Cassidy (2011) argued that it is the aggregated effects of many components that determine the efficacy of the self-regulation process, and Jansen et al. (2019) argued that there may be many different models of how to support self-regulation that may be equally valid. A key priority in supporting SRAF with academics is well designed methodologies to enable exploration of how the different elements of self-regulation come together to impact outcomes for academics and their students in specific contexts.

Individual differences are implicated in the effectiveness of SRAF (Dörrenbächer and Perels, 2016). Academics need better understanding of the role of individual differences in supporting effective self-regulation (Panadero, 2023). Further research is required on how students’ self-regulatory profiles impact their engagement in SRAF and the strategies that they use, and how best to support them, as integral to SRAF professional development. Greater focus is also needed on collaborative self-regulatory approaches. In reality, regulating oneself, being supported by others (co-regulation), and regulating together (shared regulation) are all present in many aspects of SRAF, and need consideration in training to support the most appropriate use of different regulation strategies in relation to the nature of the task.

Investing in coherent and sustained programs of SRAF professional development is important in supporting high quality and efficient assessment design that benefits academics’ and students’ mastery of assessment and feedback. In this article we have highlighted how the EAT framework provides a useful structure to facilitate conversations about how to actualize SRAF, but needs brokers on the ground that can translate the work to a specific disciplinary context as to which SRL skills are prioritized for development, and for whom. Extensive opportunities are needed for dialog to support shared understandings and effective use of SRAF pedagogies underpinned by high quality research. To support academics in implementing effective SRAF focus needs to be placed on supporting them in understanding their own self-regulatory behaviors if they are to be best placed to support their students’ acquisition of such skills.

## Data availability statement

The raw data supporting the conclusions of this article will be made available by the authors, without undue reservation.

## Ethics statement

The studies involving humans were approved by the School Research Ethics Committee of the School of Biosciences, Cardiff University, UK. The studies were conducted in accordance with the local legislation and institutional requirements. The participants provided their written informed consent to participate in this study.

## Author contributions

CE core work included Conceptualization, Writing, Formal analysis, Methodology, Resources, and Visualisation. WK core work included Data Curation, Software, Formal analysis, Visualisation, Writing. SR, SA-D, RdMG, and KD core work included the Research and Investigation process.
